# Lauflumide (NLS-4) Is a New Potent Wake-Promoting Compound

**DOI:** 10.3389/fnins.2018.00519

**Published:** 2018-08-15

**Authors:** Gianina Luca, Mojtaba Bandarabadi, Eric Konofal, Michel Lecendreux, Laurent Ferrié, Bruno Figadère, Mehdi Tafti

**Affiliations:** ^1^Faculty of Biology and Medicine, Center for Integrative Genomics, University of Lausanne, Lausanne, Switzerland; ^2^Centre Neuchâtelois de Psychiatrie, Neuchâtel, Switzerland; ^3^Department of Physiology, Faculty of Biology and Medicine, University of Lausanne, Lausanne, Switzerland; ^4^Pediatric Sleep Disorders Center, AP-HP, Robert Debre Hospital, Paris, France; ^5^AP-HP, Pediatric Sleep Center and National Reference Centre for Orphan Diseases, Narcolepsy, Idiopathic Hypersomnia and Kleine-Levin Syndrome (CNR Narcolepsie-Hypersomnie), CHU Robert-Debre, Paris, France; ^6^BioCIS, Université Paris-Sud, CNRS, Université Paris Saclay, Châtenay-Malabry, France

**Keywords:** stimulant, modafinil, rebound hypersomnia, EEG delta power, recovery sleep

## Abstract

Psychostimulants are used for the treatment of excessive daytime sleepiness in a wide range of sleep disorders as well as in attention deficit hyperactivity disorder or cognitive impairment in neuropsychiatric disorders. Here, we tested in mice the wake-promoting properties of NLS-4 and its effects on the following sleep as compared with those of modafinil and vehicle. C57BL/6J mice were intraperitoneally injected with vehicle, NLS-4 (64 mg/kg), or modafinil (150 mg/kg) at light onset. EEG and EMG were recorded continuously for 24 h after injections and vigilance states as well as EEG power densities were analyzed. NLS-4 at 64 mg/kg induced significantly longer wakefulness duration than modafinil at 150 mg/kg. Although no significant sleep rebound was observed after sleep onset for both treatments as compared with their vehicles, modafinil-treated mice showed significantly more NREM sleep when compared to NLS-4. Spectral analysis of the NREM EEG after NLS-4 treatment indicated an increased power density in delta activity (0.75–3.5 Hz) and a decreased power in theta frequency range (6.25–7.25 Hz), while there was no differences after modafinil treatment. Also, time course analysis of the delta activity showed a significant increase only during the first 2 time intervals of sleep after NLS-4 treatment, while delta power was increased during the first 9 time intervals after modafinil. Our results indicate that NLS-4 is a highly potent wake-promoting drug with no sign of hypersomnia rebound. As opposed to modafinil, recovery sleep after NLS-4 treatment is characterized by less NREM amount and delta activity, suggesting a lower need for recovery despite longer drug-induced wakefulness.

## Introduction

**Figure 1:**
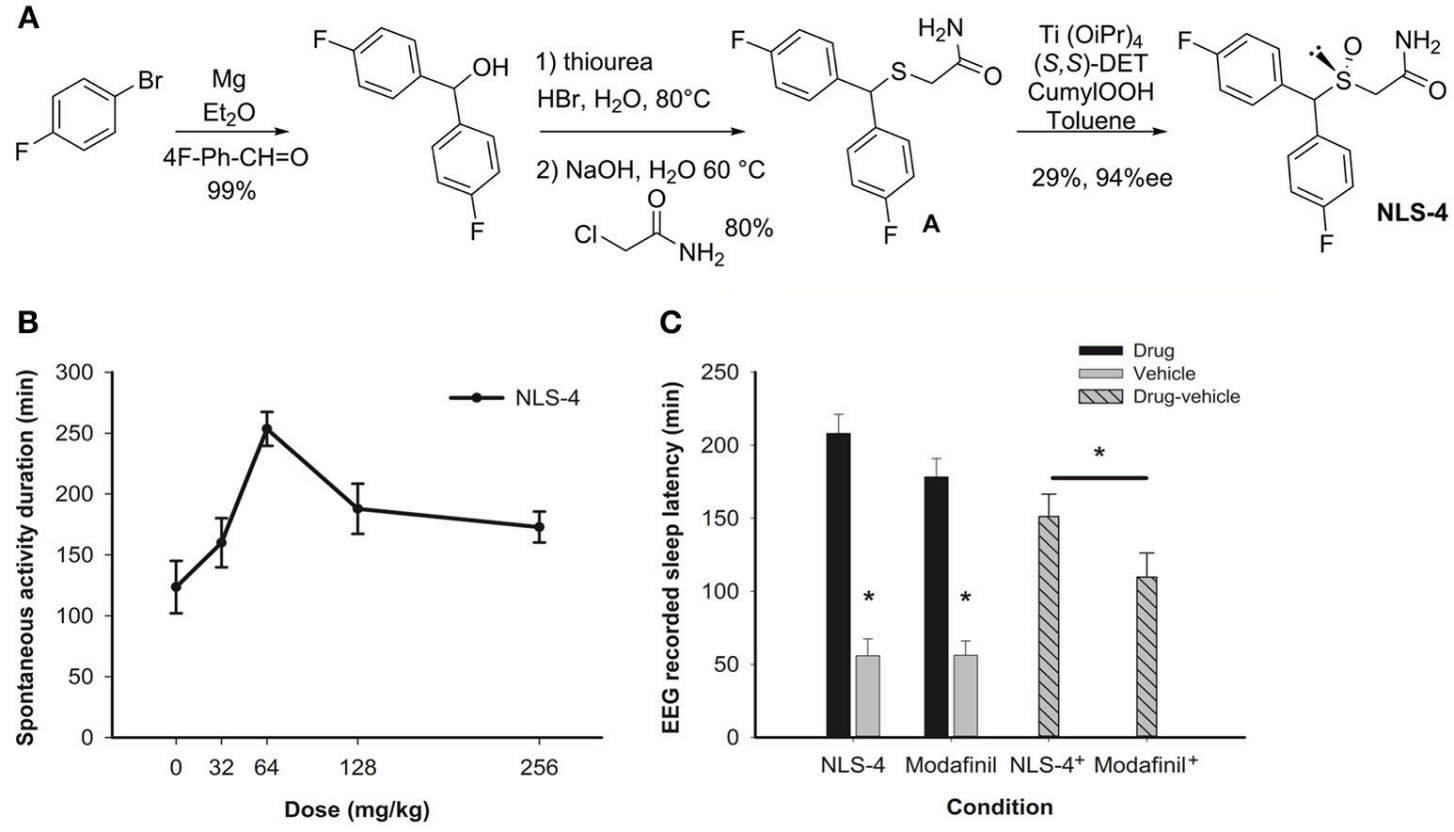
**(A)** NLS-4 was synthesized through a straightforward method involving first a Grignard addition of the desired 4-fluorophenylmagnesium bromide onto 4-fluorobenzaldehyde to provide p-fluorobenzydrol. Addition of thiourea in bromohydric medium, followed by treatment of the thiourea adduct with aqueous NaOH and alkylation with 2-chloroacetamide afforded intermediate “A.” Finally, asymmetric oxidation of thioether “A” into (*R*)-sulfoxide by using Kagan method (Pitchen and Kagan, 1984) led to expected NLS-4 compound in 23% overall yield in 4 steps (Cao et al., 2010). **(B)** Amount of spontaneous locomotor activity measured after intraperitoneal administration of increasing doses of NLS-4 at light onset (*N* = 8). **(C)** Duration of EEG recorded wakefulness after administration of 64 mg/kg of NLS-4, 150 mg/kg of modafinil, or vehicle. Both drugs significantly increased the amount of wakefulness (paierd *t*-test, *P < 0.05) but NLS-4-induced wake duration was significantly higher than that of modafinil (*t*-test, *P < *0.05*). ^+^indicates drug-vehicle value.

Excessive daytime sleepiness (EDS) is a major symptom of a wide range of sleep disorders. Clinical management of EDS includes the use of psychostimulants such as modafinil and methylphenidate. With increasing number of patients with EDS there is an increasing interest and need for wake-promoting drugs with high efficacy and low side effects. Modafinil is the gold standard stimulant for the treatment of EDS in narcolepsy and other hypersomnias (Mayer et al., 2015; Barateau et al., 2016). The mechanism of action of modafinil is still unclear, but evidence indicates that it differs from amphetamine in structure, neurochemical profile, and behavioral effects (Minzenberg and Carter, 2008). Studies suggest that although inhibition of dopamine (DA) reuptake may be a primary mechanism underlying modafinil's therapeutic actions, non-DA-dependent actions may be playing a role in its psychostimulant profile (Mereu et al., 2017). Moreover, beside its wake-promoting properties, evidence to date suggests that modafinil is well tolerated, safe, and lacking any of the euphorigenic or reinforcing properties that can lead to addiction (Ballon and Feifel, 2006). Pitolisant, a histamine H3 inverse agonist is the newest and approved second-generation stimulant with efficacy comparable to modafinil (Dauvilliers et al., 2013). Another second-generation wake-promoting drug, JZP-110 is in advanced clinical trials for the treatment of EDS in narcolepsy and sleep apnea (Bogan et al., 2015; Ruoff et al., 2016). JZP-110 is a potent dopaminergic-noradrenergic drug with efficacy higher than modafinil (Hasan et al., 2009). Lauflumide [2-((bis(4-fluorophenyl)methane)sulfinyl)acetamide] (NLS-4) was conceptualized by the R&D of Laboratoire L. Lafon but has never been developed. This bis (*p*-fluoro) ring-substituted derivative of modafinil (Figure 1A) is an original potent wake-promoting agent and produces anti-aggressive effects in rats, which modafinil does not (Patent No.:US9,637.447B2). Preliminary findings suggest that NLS-4 is a selective dopamine reuptake inhibitor, blocking (83%) dopamine transporter (DAT), higher than methylphenidate and without deleterious effects on peripheral adrenergic systems involved in hypertension (Study 100014859 CEREP 20/03/14, unpublished data).

The aim of the present study was to evaluate the wake-promoting properties of NLS-4 in mice, its effects on the following sleep and to compare its pharmacological effects on vigilance states with those of modafinil and vehicle.

## Materials and methods

### Animals

Adult male C57BL/6J mice (age: 10–11 weeks at the time of surgery; weight: 22–26 g) were purchased from Charles River. Mice were kept individually in polycarbonate cages (31 × 18 × 18 cm) with food and water available *ad libitum*, and maintained on a 12 h light–dark cycle (lights on at 09:00 h) at an ambient temperature of 24.5–25.5°C. The study protocols were approved by the Veterinary Office of the Canton of Vaud, Switzerland.

### Chemicals

NLS-4 was synthesized in 4 steps as described in Figure 1A.

We first determined the effective dose for NLS-4. C57BL/6J mice (*N* = 8) were individually housed as described above. Activity was recorded under 12:12 h light-dark cycles. To determine the NLS-4 dose that induces a robust wakefulness period, locomotor activity was monitored and recorded using infrared sensors. ClockLab software (Actimetrics) was used for both data acquisition and analyses. Five NLS-4 doses were administrated at light onset in ascending or descending order every 24 h: vehicle, 32, 64, 128, and 256 mg/kg. Sleep onset was defined as the time elapsed between the time of injection and the first inactivity episode (5 min of infrared uninterrupted inactivity). Based on dose-response analysis (Figure 1B) the dose of 64 mg/kg was chosen. For modafinil the dose of 150 mg/kg was used based on our previous work (Hasan et al., 2009).

Another set of 8 mice per treatment was randomly assigned to NLS-4 or modafinil treatment, receiving two intraperitoneal injections in a random order (drug-vehicle or vehicle-drug) at 24 h interval. After the 24 h baseline, mice received either the drug or vehicle. NLS-4 was dissolved in 10% DMSO-saline solution. As previously described (Hasan et al., 2009) modafinil was not dissolved but suspended in the saline solution by vortexing and injecting immediately each mouse. Drug solutions were freshly prepared the same day before light onset. Control solutions (vehicle) were 10% DMSO-saline solution for NLS-4 and saline for modafinil.

### Sleep recording and analysis

EEG and EMG electrodes implantation was performed under deep anesthesia as previously described (Hasan et al., 2009). EEG/EMG signals were recorded using EMBLA hardware and Somnologica-3 software (Flaga, Iceland). The animal's behavior was scored based on visual inspection of EEG and EMG signals every 4 s as wakefulness, non-rapid eye movement (NREM), or REM sleep.

The EEG spectral analysis was performed as described previously (Vienne et al., 2010). Briefly, the EEG signal (frontal-central) was subjected to a discrete Fourier transformation yielding power spectra (range: 0.25–25 Hz; resolution: 0.25 Hz; window function: hamming) for 4-s artifact-free epochs. Two mice treated with NLS-4 (*N* = 6) and one treated with modafinil (*N* = 7) were excluded from the analysis due to artifacts. Spectral composition was generated using PRANA software. The EEG delta power during NREM sleep was calculated by averaging power density in the frequency bins from 0.75 to 4 Hz (Mang and Franken, 2012). Values were normalized per mouse by expressing them as a percentage of the mean delta power over NREM sleep in the last 4 h of the baseline light period (when all mice reach the lowest 24 h level of delta power). The time course of EEG delta power in NREM sleep was calculated for the sleep following drug administration. To adjust and compensate for the different amount of NREM sleep across the light and dark cycle, NREM amounts were subdivided into intervals (12 time intervals during the light period and 6 for the dark period; see **Figure 4**) with an equal number of contributing NREM epochs for all intervals of the respective segment (e.g., the durations of NREM sleep as reported in Table 1 are divided into 12 equal segments during the light and 6 during the dark period: for NLS-4 light: 254/12 = 21 min, dark: 174/6 = 29 min on average for each time interval).

**Table 1:** Distribution of vigilance states during the 24 h after drug administration.

		**NLS-4 (*N* = 8)**	**Vehicle (*N* = 8)**	**Modafinil (*N* = 8)**	**Vehicle (*N* = 8)**	**NLS-4 vs. Modafinil**
						***P***
Sleep latency after drug injection		208.18 ± 12.99^***^^a^	55.92 ± 11.52	178.25 ± 12.59^***^^a^	56.18 ± 9.82	<0.05^b^
NREM	Light	254.41 ± 21.16^***^^c^	342.02 ± 22.67	292.73 ± 29.86^***^^c^	337.76 ± 23.06	<0.05^d^
	Dark	174.86 ± 49.90	158.50 ± 37.10	206.81 ± 32.70^**^^c^	178.94 ± 28.13	ns^d^
REM	Light	40.67 ± 6.94^**^^c^	49.84 ± 6.14	41.41 ± 10.42^**^^c^	45.74 ± 11.23	<0.05^d^
	Dark	21.88 ± 10.12^*^^c^	16.71 ± 7.67	20.95 ± 5.95^***^^c^	14.29 ± 7.85	ns^d^
Wake	Light	223.30 ± 37.57^**^^c^	273.37 ± 19.28	226.21 ± 52.35^**^^c^	285.73 ± 25.42	ns^d^
	Dark	523.25 ± 58.66	544.79 ± 44.03	490.26 ± 35.98	525.83 ± 34.54	ns^d^
NREM sleep loss	Light	−87.61 ± 36.78		−45.04 ± 19.96		<0.05^d^
	Dark	−71.25 ± 23.55		−17.17 ± 51.43		<0.05^d^
REM sleep loss	Light	−9.18 ± 3.88		−4.33 ± 4.37		<0.05^d^
	Dark	−4.23 ± 1.50		2.32 ± 6.33		<0.01

*All values are mean ± SD*.

* *P < 0.05*,

** *P < 0.01*,

*** *P < 0.001*.

a *Paired t-test*.

b *t-test*.

c *2-way repeated measures ANOVA*.

d *2-way ANOVA, ns: non-significant. NREM and REM sleep losses correspond to the differences of accumulated sleep between drugs and their respective vehicles at the end of the light and dark period*.

### Statistical analysis

The amount of drug-induced wakefulness was analyzed by paired *t*-test (drug vs. vehicle). To directly compare the between drug difference in drug-induced wakefulness the duration of vehicle-induced wakefulness was subtracted from that of the drug for each mouse and the mean durations were analyzed by *t*-test (Figure 1C). The hourly amounts of NREM sleep after drug administration were analyzed separately for each drug by a 2-way repeated measures ANOVA with factor “treatment” (drug vs. vehicle) and “time” (time points after drug injection). To directly compare the between drug differences in NREM sleep distribution the amount of vehicle-induced NREM sleep was subtracted from that of the drug for each mouse and the mean hourly values were compared by a 2-way ANOVA with factor “drug” (NLS-4 vs. vehicle) and “time” (time points after drug injection). The power densities during each vigilance states and the time course of the slow wave activity during NREM sleep were analyzed separately for each drug by a 2-way repeated measures ANOVA with factor “treatment” (drug vs. vehicle) and “frequency” (frequency bins in Hz) or “time” (time points after drug injection), respectively. To directly compare the between drug differences, values of drug condition were divided by those of vehicle for each mouse and the mean power densities were compared by a 2-way ANOVA with factor “drug” (NLS-4 vs. vehicle) and “frequency” (frequency bins in Hz), or “time” (time points after drug injection), respectively. All statistical analyses were performed with GraphPad Prism and all *post-hoc* tests were Sidak corrected for multiple comparisons.

## Results

### Vigilance states

**Figure 2:**
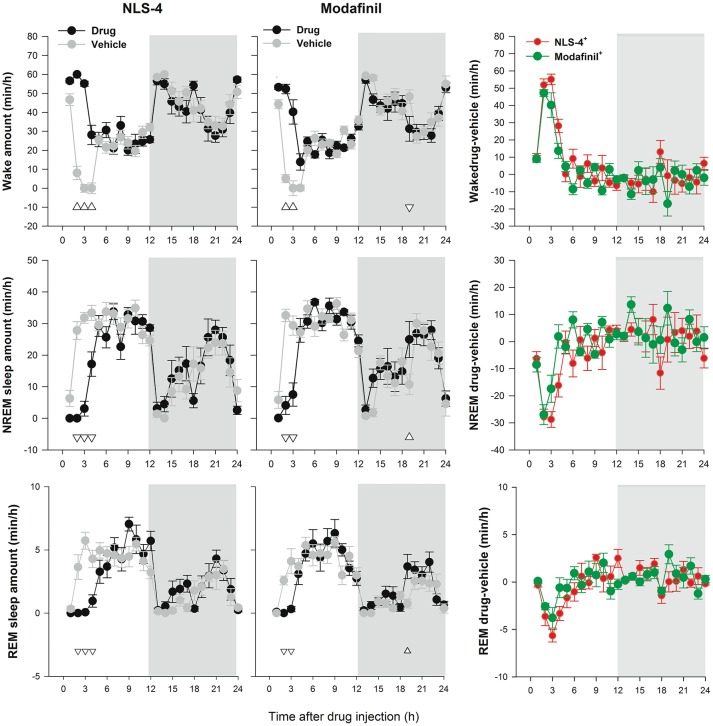
Hourly distribution of vigilance states after NLS-4, modafinil, and vehicle administration (mean±SEM). After initial suppression of NREM sleep by both drugs [2-way repeated measures ANOVA, NLS-4: “treatment” *p* < 0.0001, *F*_(1, 7)_ = 73.25, “hour”, *p* < 0.0001, *F*_(23, 161)_ = 17.21, interaction, *p* < 0.0001, *F*_(23, 161) =_ 4.16; modafinil: “treatment” *p* > 0.38, *F*_(1, 7)_ = 0.89, “hour” *p* < 0.0001, *F*_(23, 161)_ = 20.18; interaction, *p* < 0.0001, *F*_(23, 161)_ = 5.10], the distribution of NREM sleep during recovery is very similar to that after vehicle indicating the absence of sleep rebound (hypersomnia). Nevertheless, comparisons between the two drugs indicated significantly less NREM sleep after NLS-4 administration as compared to modafinil [2-way ANOVA, drug effect: *F*_(1, 14)_ = 7.31, *p* < 0.02]. REM sleep is also suppressed during the first hours after drug injection while wakefulness is increased. Upward and downward triangles indicate significant increase or decrease, respectively. Shaded areas indicate the dark period. ^+^indicates drug/vehicle ratio.

Both NLS-4 (*N* = 8) and modafinil (*N* = 8) increased the duration of drug-induced wakefulness compared with vehicle (Figure 1C) (paired *t*-test between “drug” and “vehicle” condition, *p* < 0.05, Table 1). Subtracting the vehicle-induced from that of drug-induced wakefulness indicated that NLS-4 administration resulted in an overall longer wakefulness duration (151.18 ± 15.33 min for NLS-4 and 109.67 ± 16.59 min for modafinil, *p* < 0.05, *t*-test, Figure 1C). Neither NLS-4 nor modafinil induced hyper locomotor, stereotypic activity, or abnormal behavior (by direct observation, infra-red and video recording). The distribution of vigilance states after sleep onset (defined as 3 consecutive epochs of NREM sleep or 12 s) was affected in both groups. NREM sleep amount for NLS-4 was significantly lower, and NLS-4-treated mice had overall less NREM sleep as compared with modafinil-treated group (Figure 2). The recovery of lost NREM sleep (NREM accumulation minus vehicle) at the end of the light period (12 h after drug administration) indicated a deficit of 87.61 ± 36.78 min for NLS-4 and of 45.04 ± 19.96 min for modafinil-treated mice. For modafinil-treated group the recovery of lost NREM sleep further continued to the dark period, leading into a significantly larger amount of accumulated NREM sleep at the end of the 24 h recording (Table 1).

For both NLS-4 and modafinil-treated groups, REM sleep amount was decreased during the light and increased during the dark recovery period, as compared to vehicle (Figure 2, Table 1). The analysis of vigilance states bout duration and number indicated no significant differences between drugs or between drugs and vehicles.

### EEG spectral composition after the drug administration

The EEG spectral composition did not differ at baseline or during vehicle administration between the two drugs. We compared NREM, REM and Wake relative power densities to evaluate the differences between vehicle and drug (2-way repeated measures ANOVA, factors “drug,” “bin (Hz),” and their interaction). Also, the spectral composition was compared between the two drugs. A similar analysis was also performed for absolute power densities.

#### NREM sleep

**Figure 3:**
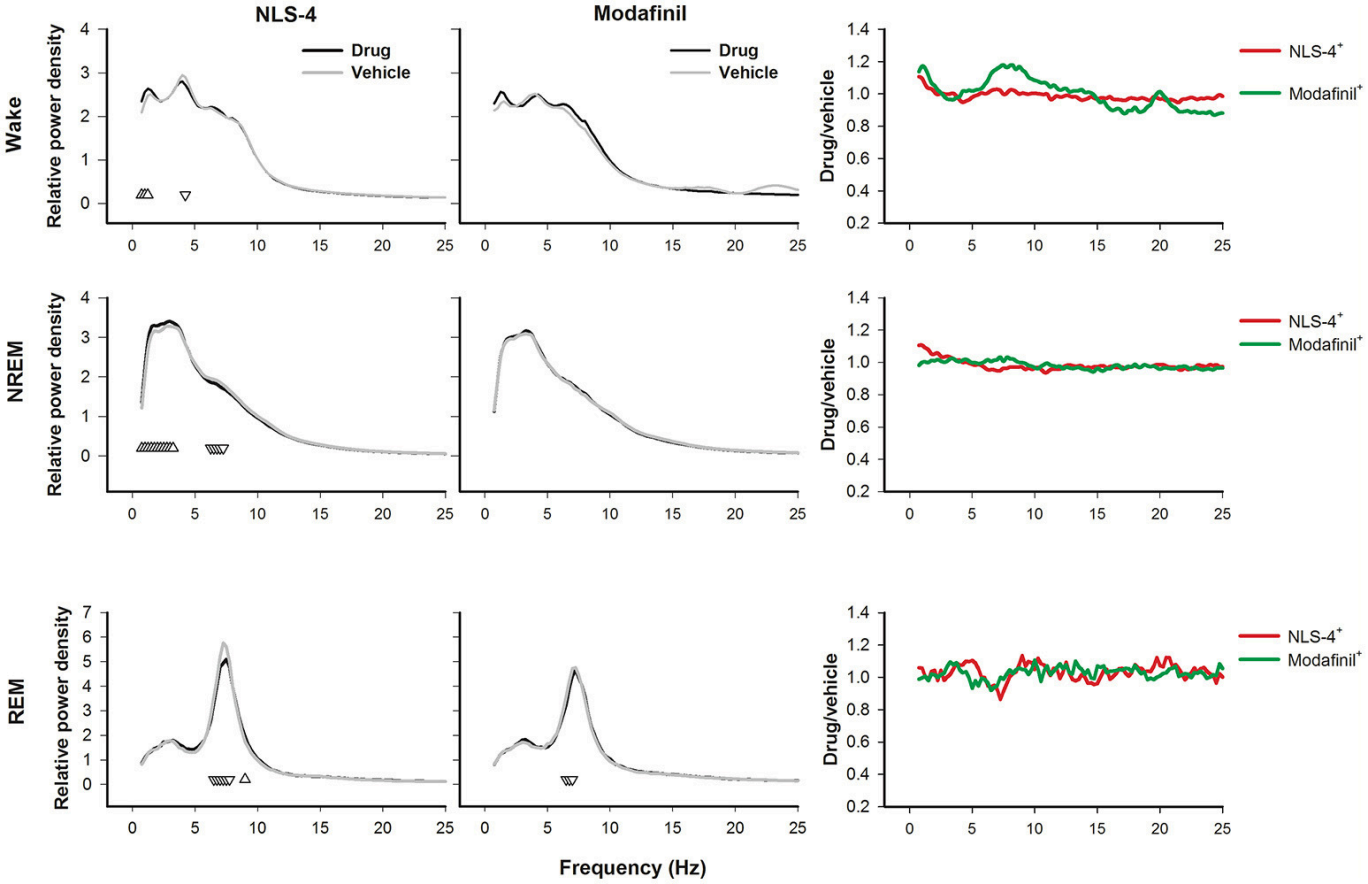
Relative spectral profiles of wake, NREM and REM sleep after drug-induced wakefulness (from sleep onset till the end of the 24 h recording). No changes were observed between modafinil and vehicle while NLS-4 showed increased power densities in delta frequency range [2-way repeated measures ANOVA, “treatment” *p* > 0.65, *F*_(1, 5)_ = 0.22, “bin” *p* < 0.0001, *F*_(97, 485)_ = 133.60, interaction *p* < 0.0001, *F*_(97, 485)_ = 6.66]. Additionally, theta power density was decreased during NREM and REM sleep under NLS-4 treatment [2-way repeated measures ANOVA, “treatment”, *p* > 0.16, *F*_(1, 5)_ = 2.57, “bin” *p* < 0.0001, *F*_(97, 485)_ = 736.60, interaction *p* < 0.0001, *F*_(97, 485)_ = 4.28; *multiple comparisons: p* < 0.05 for: 6.5–7.75 Hz, with drug < vehicle]. Low delta power was increased during wakefulness after NLS-4 treatment while no changes were observed after modafinil [2-way repeated measures ANOVA, NLS-4: “treatment” *p* > 0.28, *F*_(1, 5)_ = 1.45, “bin” *p* < 0.0001, *F*_(97, 485)_ = 64.03, interaction *p* < 0.01, *F*_(97, 485)_ = 1.55, *multiple comparisons: p* < 0.05 for: 0.75–1.25 Hz, with drug>vehicle and 4.25 Hz, with drug < vehicle]. Upward and downward triangles indicate significant increase or decrease in power, respectively.

NLS-4 induced an increase in 0.75–3.25 Hz frequency range, and lower relative power in 6.25–7.25 Hz frequency range (Figure 3). No significant differences in overall spectral composition were identified for modafinil-treated mice. Analysis of absolute power densities confirmed that only NLS-4 increased delta power. Between drug comparisons did not reveal any significant drug or interaction effect (Supplementary Figure 1).

#### REM sleep and wake

Only NLS-4 induced a decrease in relative theta power densities during REM sleep (Figure 3). A similar decrease was also found for the absolute power densities (Supplementary Figure 1).

NLS-4 induced a significant increase in the relative low delta power during wakefulness while modafinil had no effect (Figure 3). Similarly, NLS-4 treatment induced an increase in absolute power in low delta frequencies during wakefulness, while modafinil-treated mice showed lower absolute power at 8.25 Hz only (Supplementary Figure 1).

#### EEG delta power during NREM sleep

**Figure 4:**
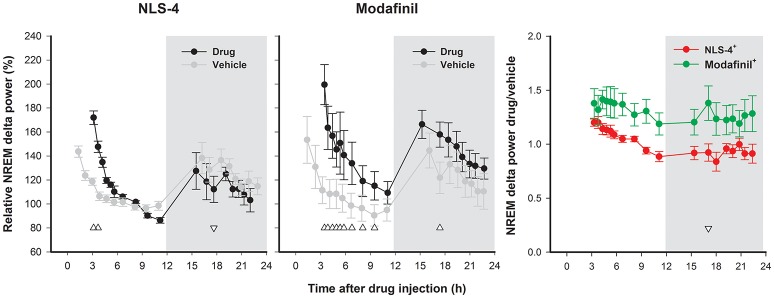
Time course of slow-wave (delta) activity (0.75–4 Hz) during NREM sleep following drug-induced wakefulness. Delta power is increased during the first 2 time intervals of sleep after NLS-4 [2-way repeated measures ANOVA, “treatment” *p* > 0.9, *F*_(1, 5)_ = 0.01, “hour” *p* < 0.001, *F*_(17, 85)_ = 11.79, interaction *p* < 0.001, *F*_(17, 85)_ = 5.04] and during the first 9 time intervals after modafinil administration [2-way repeated measures ANOVA, “treatment” *p* < 0.05, *F*_(1, 6)_ = 9.35, “hour” *p* < 0.001, *F*_(17, 102)_ = 17.88, interaction *p* < 0.05, *F*_(17, 102)_ = 2.04]. Comparison between drugs indicates an overall increased spectral power after modafinil administration [2-way ANOVA, “drug” *p* < 0.05, *F*_(1, 11)_ = 5.23, “hour” *p* < 0.001, *F*_(17, 187)_ = 4.39, interaction *p* = ns, *F*_(17, 187)_ = 0.93]. Upward and downward triangles indicate significant increase or decrease in delta power, respectively. Shaded areas indicate the dark period.

Sleep following the drug-induced wakefulness showed a significant increase in delta power. Delta power was increased only for the first 2 time intervals for NLS-4-treated mice (Figure 4), while modafinil treatment resulted in increased delta power for the first 9 time points and time point 12. The overall difference in NLS-4 treated mice was significantly lower than in modafinil-treated ones (Figure 4).

#### Spectral composition of drug-induced wakefulness

The comparison between drug-induced and vehicle-induced wakefulness showed only an increase in low delta power [2-way repeated measures ANOVA, NLS-4: “treatment” *p* > 0.1, *F*_(1, 5)_ = 2.5, “bin” *p* < 0.001, *F*_(97, 485)_ = 46.23, interaction *p* < 0.05, *F*_(97, 485)_ = 1.31, modafinil: “treatment” *p* > 0.7, *F*_(1, 6)_ = 0.07, “bin” *p* < 0.001, *F*_(97, 582)_ = 39.25, interaction *p* < 0.01, *F*_(97, 582)_ = 1.46, *multiple comparisons*: *p* < 0.05 for 0.75 Hz with drug>vehicle].

## Discussion

We compared vigilance states and spectral power densities during and after administration of NLS-4 and modafinil in mice. NLS-4 was found to be a highly potent wake-promoting drug with less than half of the modafinil dose resulting in significantly longer wakefulness. No significant changes in behavior (signs of hyper locomotor activity) were found either under NLS-4 or modafinil. Rebound hypersomnia (over compensation), typically found with amphetamine or methyphenidate (Gruner et al., 2009; Hasan et al., 2009), was not observed for either drugs. Nevertheless, more sleep was found after wake-induced period by modafinil as compared to NLS-4. Spectral analysis of wakefulness during and sleep after drug-induced wakefulness revealed that both drugs increased EEG power densities within the slow frequencies (< 4 Hz) but the increase by NLS-4 was significantly lower than modafinil despite the fact that NLS-4 induced a longer wakefulness amount. Although NLS-4 is a derivative of modafinil, our results indicate that not only is it more potent than modafinil at the doses tested but also wakefulness induced by NLS-4 is compensated by less NREM sleep as well as delta power.

### Drug effects on wakefulness amount

Our dose-response study based on spontaneous locomotor activity indicated a substantial increase in NLS-4-induced wakefulness up to 64 mg/kg but no further increment at 128 and 256 mg/kg. We previously reported that modafinil increased wakefulness in a linear manner between 100 and 300 mg/kg (Hasan et al., 2009). The dose of 64 mg/kg was chosen for NLS-4 because this dose induced ~4 h of wakefulness (Figures 1C, 2) similar to our previous report with modafinil at 150 mg/kg (Hasan et al., 2009). Sleep recordings indicated a large increase in wakefulness during the first 4 h after both NLS-4 and modafinil administration as compared to vehicle and NLS-4 at 64 mg/kg induced significantly longer wakefulness duration as compared to modafinil at 150 mg/kg. These results indicate that NLS-4 is more potent wake-promoting drug than modafinil at the doses tested.

### Drug effects on EEG during induced wakefulness

One important aspect of any stimulant is the quality of the induced wakefulness. Our direct observations (visually and by video) did not reveal any hyperactivity under NLS-4 or modafinil. We next analyzed the EEG during drug-induced wakefulness. As compared with vehicle, waking induced by NLS-4 as well as modafinil showed a significant increase in low delta activity only. This finding is consistent with the drug-induced long wakefulness duration. Since no other significant changes were observed, we conclude that both drugs produce similar and normal wakefulness.

### Drug effects on recovery sleep

The effects of both NLS-4 and modafinil disappeared after 4 and 2 h and from this point on there were no significant differences between the drug and the vehicle condition (except a slight increase in NREM sleep at hour 19 under modafinil). This indicates that none of these drugs induce rebound hypersomnia. Rebound hypersomnia is a well-established side effect of the classical stimulants such as amphetamine, methamphetamine, and phentermine (Gruner et al., 2009; Hasan et al., 2009). It was suggested that this side effect is due to increased release of cathecholamines, especially dopamine, while dopamine reuptake blockers do not present such an undesirable side effect (Gruner et al., 2009). Nevertheless, cocaine, bupropion, and methylphenidate (dopamine active transporter inhibitors) also induce rebound hypersomnia, suggesting that other mechanisms (such as pharmacokinetic) are involved (Gruner et al., 2009). Other stimulants such as mazindol and JZP-110 are lacking the rebound hypersomnia and must act as dopamine transporter inhibitors (Gruner et al., 2009; Hasan et al., 2009). It was also suggested that some stimulants such as cocaine produce late hypersomnia (12–24 h after administration) (Dugovic et al., 1992). NLS-4 not only did not induce late NREM sleep rebound but even induced a decrease in the middle of the dark period with a significant decrease in delta power (18 h after drug administration), while modafinil induced a significant increase in NREM sleep at hour 19 (7 h into the dark period) as compared with vehicle. Comparison of recovery sleep between the two drugs revealed significantly less NREM sleep after NLS-4 administration (Figure 2), suggesting that less NREM sleep is accumulated (or compensated) during first hours of recovery after NLS-4 as compared to modafinil.

### Drug effects on the recovery sleep EEG

Modafinil did not change any aspects of the waking EEG spectral powers during the recovery period while NLS-4 induced increased low delta and decreased high delta (Figure 3). Power densities within the delta range were significantly increased during recovery NREM sleep after NLS-4 while theta power density was decreased. Modafinil did not induce any significant changes. Overall, NLS-4 significantly increased power densities when compared to vehicle, strongly suggesting that recovery after NLS-4 occurred principally through an increase in sleep intensity. Only NLS-4 induced decreased power densities in high theta range (6.5–7.25 Hz) during REM sleep, as compared with vehicle. The significance of the changes in the theta range during both NREM and REM sleep remains unknown.

The time course analysis of the delta activity during NREM sleep (an index of sleep homeostasis) indicated a significant increase (during the first 2 time intervals of recovery sleep) after NLS-4 and more after modafinil (first 9 time intervals of recovery sleep). Interestingly, mice slept less during this period after NLS-4 confirming that recovery after NLS-4-induced wakefulness occurred with less NREM amount and less delta activity. Another interesting observation is that although animals under NLS-4 stayed awake longer, delta activity was increased during a longer period in modafinil treated animals after sleep onset, suggesting that recovery occurred faster after NLS-4.

In summary, NLS-4 is a potent stimulant with different effects than its parent compound modafinil (Rambert et al., 1986) and most other stimulants in terms of potency and effects on sleep rebound and the sleep EEG.

## Ethics statement

This study was carried out in accordance with the recommendations of the Swiss Federal Food Safety and Veterinary Office. The protocol was approved by the Veterinary Office of the Vaud State, Lausanne.

## Author contributions

MT, EK, and ML designed the study. GL conducted the experiments. MT, GL, and MB analyzed the data. LF and BF synthetized the NLS-4 compound. All authors contributed to the draft and the final version of the manuscript.

### Conflict of interest statement

MT, EK, ML and BF received compensation from private or publicly owned organizations for serving as an advisory or scientific board member or as an invited speaker. The remaining authors declare that the research was conducted in the absence of any commercial or financial relationships that could be construed as a potential conflict of interest.

## Supplementary material

Supplementary Figure 1 Absolute spectral profiles of wake, NREM and REM sleep after drug-induced wakefulness. NLS-4 showed increased power densities in delta frequency range during wakefulness and NREM sleep and decreased power densities in theta frequency range [NLS-4: 2-way repeated measures ANOVA, “treatment” *p* > 0.11; *F*_(1, 5)_ = 3.66, “bin” *p* < 0.0001, *F*_(97, 485)_ = 46.43, interaction *p* < 0.0001*, F*_(97, 485)_ = 5.80*; multiple comparisons: p* < 0.05 for: 0.75–4.00 Hz, with drug>vehicle]. Low delta power was increased during wakefulness after NLS-4 treatment [2-way repeated measures ANOVA, “treatment” *p* > 0.5; *F*_(1, 5)_ = 0.51, “bin” *p* < 0.0001, *F*_(97, 485)_ = 24.20, interaction *p* < 0.001, *F*_(97, 485)_ = 1.59; *multiple comparisons*: *p* < 0.05 for: 0.75–1.25 Hz, with drug>vehicle] while modafinil induced lower absolute power at 8.25 Hz [2-way repeated measures ANOVA, “treatment” *p* > 0.39, *F*_(1, 6)_ = 0.84, “bin” *p* < 0.0001, *F*_(97, 582)_ = 20.90, interaction *p* < 0.01, *F*_(97, 582)_ = 1.43; *multiple comparisons*: *p* < 0.05 for 8.25 Hz, with drug>vehicle]. Upward and downward triangles indicate significant increase or decrease in power, respectively.
